# Genome Recombination-Mediated tRNA Up-Regulation Conducts General Antibiotic Resistance of Bacteria at Early Stage

**DOI:** 10.3389/fmicb.2021.793923

**Published:** 2022-01-20

**Authors:** Huiying Fang, Guandi Zeng, Wei Gu, Yubin Wang, Jing Zhao, Tingkai Zheng, Lina Xu, Yutong Liu, Jinning Zhang, Xuesong Sun, Gong Zhang

**Affiliations:** Key Laboratory of Functional Protein Research of Guangdong Higher Education Institutes, Institute of Life and Health Engineering, Jinan University, Guangzhou, China

**Keywords:** tRNA, translation, antibiotic resistance, recombination, oxidative stress

## Abstract

Bacterial antibiotic resistance sets a great challenge to human health. It seems that the bacteria can spontaneously evolve resistance against any antibiotic within a short time without the horizontal transfer of heterologous genes and before accumulating drug-resistant mutations. We have shown that the tRNA-mediated translational regulation counteracts the reactive oxygen species (ROS) in bacteria. In this study, we demonstrated that isolated and subcultured *Escherichia coli* elevated its tRNAs under antibiotic stress to rapidly provide antibiotic resistance, especially at the early stage, before upregulating the efflux pump and evolving resistance mutations. The DNA recombination system repaired the antibiotic-induced DNA breakage in the genome, causing numerous structural variations. These structural variations are overrepresented near the tRNA genes, which indicated the cause of tRNA up-regulation. Knocking out the recombination system abolished the up-regulation of tRNAs, and coincidently, they could hardly evolve antibiotic resistance in multiple antibiotics, respectively. With these results, we proposed a multi-stage model of bacterial antibiotic resistance in an isolated scenario: the early stage (recombination—tRNA up-regulation—translational regulation); the medium stage (up-regulation of efflux pump); the late stage (resistant mutations). These results also indicated that the bacterial DNA recombination system and tRNA could be targeted to retard the bacterial spontaneous drug resistance.

## Introduction

Bacterial antibiotic resistance (AR) is a major threat on health. The number of newly developed antibiotics is rapidly declining over the years (Shlaes et al., 2013). In sharp contrast, bacteria evolve AR shortly after the application of newly developed antibiotics (Centers for Disease Control and Prevention, 2013), emphasizing an alarming “antibiotic crisis” (Ventola, 2015a,b). Studies have shown that numerous AR genes exist in nature for centuries already, providing AR by horizontal transfer (Martinez, 2008; Sommer et al., 2010; Nesme and Simonet, 2015). However, this theory is difficult to explain the fact that bacteria quickly evolve resistance against non-natural, fully artificially synthesized antibiotics (Centers for Disease Control and Prevention, 2013), for which the specific natural AR genes rarely exist. This indicates a general and intrinsic mechanism of bacteria to resist any antibiotics without any heterologous AR gene input.

Two major types of spontaneous AR mechanisms have been revealed without any heterologous AR gene input: (a) mutation or modification of the antibiotic targets; (b) increasing efflux or reducing membrane permeability to limit the cellular antibiotic concentration (Blair et al., 2015; Yelin and Kishony, 2018). However, these mechanisms take effect quite slowly. For example, continuous antibiotic pressure leads to accumulation of genomic point mutations of *Escherichia coli* starting from 2 to 5 days, causing non-synonymous mutations and thus alters the proteins, spontaneously evolving AR without heterogenous genes (Toprak et al., 2012). If the bacteria cannot withstand the damage caused by the drugs at the beginning, they have no chance to develop specific resistance (e.g., genetic mutations). These facts indicate that bacteria have an intrinsic and general mechanism to evolve early AR independent of point mutations or specific AR genes.

It is known that most antibiotics facilitate generation of reactive oxygen species (ROS) in cells, which damages nucleic acids and proteins. Therefore, counteracting ROS is a must to resist most antibiotics. Our previous work demonstrated that the bacteria almost halt the translation when encountering oxidation reagents by rapidly degrading tRNAs to prevent production of damaged proteins. After adapted to the oxidative stress, the tRNA levels were resumed or even higher than normal state, and thus maintained the protein production. Overexpressing tRNAs facilitates the adaptation under oxidative stress and makes the bacteria growing better under ciprofloxacin (Zhong et al., 2015). Therefore, we posit that the abovementioned the tRNA regulation as intrinsic and general mechanism, for early AR. Revealing such a mechanism in detail would help us to understand the robustness of bacteria and find an alternative solution against AR.

## Materials and Methods

### Bacterial Strains

*Escherichia coli* strains BW25113 and BW26355 (Δ*recA* version of BW25113) were purchased from the Coli Genetic Stock Center of Yale University. The reference genome sequence NZ_CP009273.1 and its annotation (downloaded from NCBI) were used in the bioinformatics.

### Subculturing Bacteria in Antibiotics

The sensitive bacteria (the BW25113 strain) were cultured in the LB medium from a single colony as the generation 0. A new generation of culture was inoculated at 1:500 ratio and cultured at 37°C for 12 h with the presence of the antibiotics at 1/2 minimal inhibition concentration (MIC) (Watanakunakorn, 1988), MIC was determined according to the literature (Andrews, 2001). In brief, the bacterial culture was added into 6 wells in a 48-well plate with different antibiotic concentrations. After cultured at 37°C, shaked at 200 rpm for 12 h, the antibiotic concentration in the well, in which the bacteria OD600 nm was right under 0.1, was recorded as the MIC. The MIC was measured for each generation every 12 h. The population was subjected for sequencing as a pool for each generation.

### Competition Assay

The *E. coli* BW25113 transformed with pBAD33 and pRIL plasmid, respectively (Zhong et al., 2015), were mixed at 1:1 ratio. The mixture was subcultured in LB medium with the presence of 100 μg/mL chloramphenicol and ciprofloxacin at 1/2 MIC. As a control, the mixture was subcultured in LB medium with the presence of chloramphenicol but without ciprofloxacin. For each generation, the plasmids were extracted from the bacterial culture and resolved in agarose gels.

### Overexpressing Assay

The fragment of three gly-tRNA gene tandem duplication was synthesized and inserted into pRIL between SpeI/FspI restriction sites, replacing the original three tRNA genes of the pRIL. The original tRNA genes were removed from pRIL by homologous recombination, as negative control. Vectors were then transformed into wild-type BW25113. The positive clones were screened by chloramphenicol resistance. The transformed bacteria were subcultured under ciprofloxacin at 1/2 MIC (25 ng/ml). The OD and growth curves were recorded at 1/2 MIC using BacOD-24 instrument (24-well version, Chi-Biotech) for 14 h.

### RT-qPCR Assay

Total RNA was extracted by TRIzol (Invitrogen). Reverse transcription was performed with random primers by HiScript II Q RT SuperMix (Vazyme, R233). Real-time PCR was performed with Ssofast EvaGreen Supermix (Bio-Rad). The primers used: tRNA-glyGCC F: GAATAGCTCAGTTGGTAGAGCAC, tRNA-glyGCC R: GAG ACTCGAACTCGCGACC; 5S rRNA F: CCCATGCCGAAC TCAGAAGT, 5S rRNA R: CTGGCAGTTCCCTACTCTCG. All real-time PCR was performed in triplicate. The 5S rRNA was used as internal reference. Ct values were analyzed statistically using Student’s *t*-test.

### Quantitative Gel Electrophoresis

Equal amounts of RNA samples of a selected generation were loaded and subjected to electrophoresis, the agrose electrophoresis run 150 mV for 30 min on 3% agrose gel, the polyacrylamide electrophoresis run 130 mV for 30 min on 15% polyacrylamide gel. The grayscale of rRNA and tRNA bands was measured and analyzed by photoshop.

### Whole Genome Sequencing

The genomic DNA was extracted using HiPure Bacterial DNA extraction kit (Magen). The genomic DNA was sonicated into ∼300 bp fragments. The whole-genome sequencing library was constructed using MGIEasy DNA library construction kit (MGI) following the manufacturer’s instructions. The libraries were sequenced on an MGISEQ-2000 sequencer at PE100 mode.

For single nucleotide variation (SNV) analysis, the reads were mapped to reference genome using FANSe3 with the parameters -E3 -S13 –indel. The SNV were detected using the previously published and experimentally validated method (Wu et al., 2014).

For structural variation (SV) analysis, each read was trimmed into 26 nt reads. To balance the throughput of each generation, 57.4 M reads were used in the dataset of each generation. The reads were mapped to the reference genome using FANSe3 with the parameters -E3 -S13 –unique. The entire genome was divided into 600 nt bins. The uniquely mapped reads were merged according to the bins. The reads, whose two ends were mapped far away (more than 1,000 nt apart) or mapped to the same direction (indicating inversion) were counted as “SV reads.” Fisher exact test was performed for each bin with the null hypothesis that no SV happens.

Nanopore single-molecule long-read sequencing was performed according to the manufacturer’s protocol. Base calling was conducted using Guppy software with default parameters. Reads were aligned to BW25113 reference genome using Minimap2 software. Structural variations were called using SVIM software with default parameters.

### Transcriptome Sequencing

The bacterial cells were harvested by centrifugation at 4°C 4,000 × g for 5 min. The cell pellet was resuspended and washed using PBS. The cells were treated using 1.25 mg/mL lysozyme at 4°C for 10 min and collected by centrifugation at 5,000 × g for 5 min. The pellet was dissolved in 1 mL Trizol reagent, and the RNA was extracted using Trizol method. The rRNA was removed using the RiboX rRNA removal kit (Chi-Biotech) following the instructions. The RNA library was constructed using the MGIEasy mRNA library prep kit V2 and sequenced on an BGISEQ-500 sequencer at SE50 mode.

The gene expression levels were quantified using the Chi-Cloud NGS analysis platform.^1^ In brief, the reads were mapped to the reference genome using FANSe3 with the parameters -E3 –indel. The gene expression was measured using rpkM method (Mortazavi et al., 2008). The log10 rpkM values were subjected to the correlation, PCA, and clustering analyses. Gene ontology overrepresentation analysis was performed using PANTHER-DB.^2^ Significance was considered when FDR < 0.01.

### tRNA Sequencing

We developed a method to specifically sequence tRNAs in BGISEQ/MGISEQ sequencers. The sequences of adapters and primers are: Y5: 5′-TAAGACCGCTTGGCCTCCGACT TACTGGATACTGG+rN (rN = equal mixture of rA, rT, rG, rC), Y3: 5′-GTATCCAGTN_16_AAGTCGGATCGTAGCCATG (N_16_ = 16 random nucleotides), barcode primer: 5′-TGTGAGCCAAGGAGTTGTAGTGGGGATTTGTCTTCCTA AGACCGCTTGGCCTCCGACT, adapter primer: 5′-GAACGACATGGCTACGATCCGACTT (5′-phosphorylated). These adapters and primers were mixed at 1:1 ratio. Starting from 6 μg total RNA of *E. coli*, 60 ng human tRNA gel-recovered from total RNA of A549 cell line was added as spike-in. The tRNAs were deaminoacylated in 0.2 M pH = 9.5 Tris-HCl buffer at 37°C for 45 min. The RNA was then purified using HiPure RNA pure micro kits (Magen). 200∼300 ng RNA was mixed with the primers. Ligation was conducted using T4 double stranded RNA ligase 2 (NEB) following the manufacturer’s instructions. Reverse transcription was performed using the SuperScript III Reverse Transcriptase (Invitrogen) at 55°C for 45 min and 70°C for 15 min. The library was PCR amplified using the barcode primer and the adapter primer for 20 cycles. The library was sequenced on a BGISEQ-500 sequencer at SE100 mode.

The *E. coli* and human tRNA reference sequences were downloaded from the gtRNAdb.^3^ The reads were mapped to the reference sequences using BLAST (run in local server). The tRNA read counts were normalized according to the spike-in tRNA read counts.

## Results

### Bacteria Evolve Antibiotic Resistance Without Detectable Mutations in Protein-Coding Regions

**FIGURE 1:**
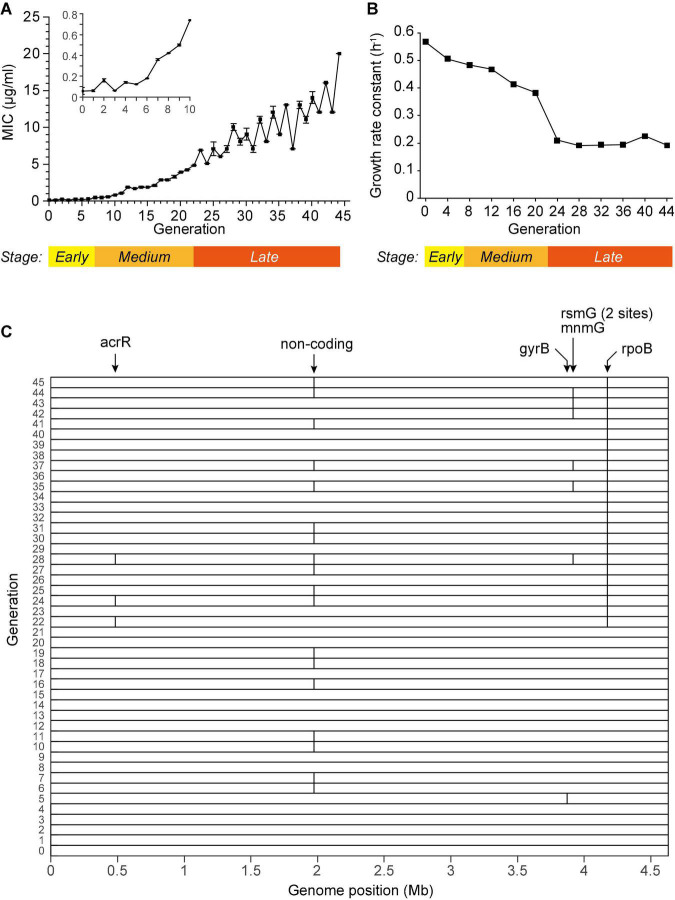
Subculturing *E. coli* BW25113 in the sub-lethal concentration of CIP. **(A)** The MIC of each generation. **(B)** The growth rate constant measured every 4 generations. **(C)** Single nucleotide mutations detected in the WGS of each generation, marked as vertical bars. The affected genes were marked on the top. Some closely located mutations are not distinguishable in the figure due to the limited resolution. Detailed information of each mutation is listed in Supplementary Table 2.

We cultured *Escherichia coli* BW25113 in LB medium under ciprofloxacin (CIP) at a concentration of 1/2 MIC (minimal inhibition concentration) in a continuous subculture way. New generation of culture is inoculated subsequently from previous culture every 12 h. In the entire process, *E. coli* was cultured in sterile flasks to avoid heterogeneous genes. The MIC gradually increased over 330-fold after 45 generation subcultures (Figure 1A). The growth rates decreased during the subculturing and almost reached constant after the 24th generation (Figure 1B). Visually, these phenotypes divide the entire subculturing into three distinct stages: (a) The early stage (before the 7th generation), where the MIC instantly increased but fluctuate around 0.1∼0.2 μg/ml, and the growth rate remarkably decreased; (b) the medium stage (approximately generation 8∼21), where the MIC steadily increased, and the growth rate steadily decreased; (c) the late stage (from the 22nd generation), where the MIC increased in a zigzagged manner to more than 100-fold compared to the sensitive strain, and the growth rate maintained at an almost constant level, less than half of which of the sensitive strain. These three stages are marked on Figures 1A,B. The stage-wise evolution of the AR indicated that the bacteria respond to the CIP using distinct mechanisms in each stage.

We first speculated that the AR was endowed by mutations in genes. To screen the non-synonymous mutations that may associate with the spontaneous drug resistance, we performed whole genome sequencing (WGS) for each generation, yielding more than 200 × sequencing depths and more than 98.98% coverage (Supplementary Table 1). SNV calling were performed according to the published method, whose accuracy and sensitivity were validated via massive Sanger sequencing (Wu et al., 2014). Only 7 SNVs were identified from the subcultured bacteria, and 6 of them were in the CDS (Figure 1C and Supplementary Table 2). Among them, only one mutation in the gene *rpoB* was persistently observed from the 22nd generation, which is known to endow resistance (Barrett et al., 2019). However, all the other mutations were sporadically detected in a few generations. For example, the drug target of CIP, the *gyrB* gene, was found mutated only in the 5th generation and not in any other generations. Thus, this mutation is unlikely to explain the resistance. This result showed that no meaningful mutations in coding genes were detected in the early stages.

### Translation Is Up-Regulated as a Response Against Antibiotics at an Early Stage

**FIGURE 2:**
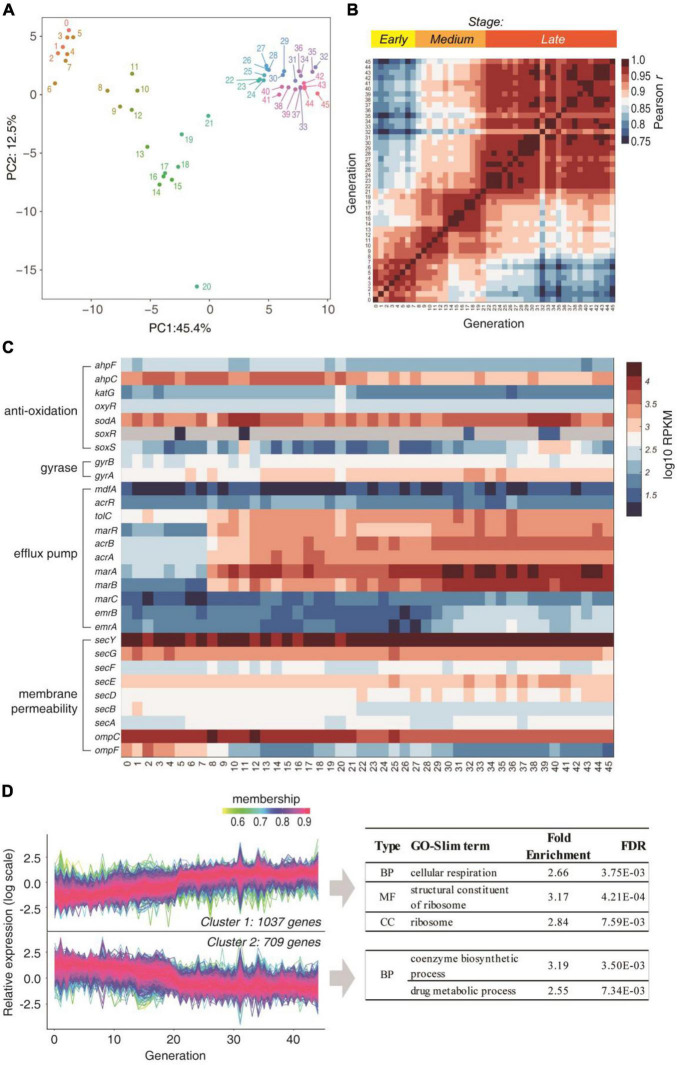
Transcriptome changes during the subculture. **(A)** PCA analysis of the gene expression profiles of each generation. Numbers denote the generation. **(B)** Mutual Pearson correlation coefficients of log RPKM values between each two generations. **(C)** Temporal change of gene expression of genes that are involved in traditional AR mechanisms. **(D)** Expressed genes were clustered into two clusters according to the expression trend over the generations. The GO-Slim terms with fold enrichment > 2 and FDR < 0.01 were filtered and listed.

Since no mutations were associated with the AR at early stages, we next performed RNA-seq for each generation to analyze the temporal change of the transcriptome in the presence of CIP. The PCA plot of the gene expression levels can be clustered into three distinct stages: the early stage (original and generations 1∼7), the medium stage (generations 8∼21), and the late stage (generations 22∼45) (Figure 2A). The mutual Pearson correlation matrix also confirmed this (Figure 2B). This corresponds to the three stages illustrated in Figures 1A,B.

We then looked deeper into the temporal expression profiles of traditional resistance genes (Figure 2C). Under the constant presence of CIP, which induces cellular ROS, the antioxidation system SoxR and SoxS did not increase over the generations. The DNA gyrases, which are the target of CIP, also did not change significantly over the generations. The only obvious trend is the elevated expression of *acrA, acrB, marA, marB*, and *marR*, which are associated with drug efflux. Nevertheless, these genes did not overexpress before the 8th generation, indicating that they are not the cause of AR at the early stage. Moreover, the genes regulating outer membrane permeability and most drug efflux genes (e.g., *marC, emrA, emrB, lamb, phoE*, etc.) were not up-regulated implying drug uptake and efflux were not altered. These results showed that the traditional resistance mechanisms cannot explain the early stage response.

Among the 2,429 genes that were quantified in all generations, 1,746 genes showed significant trends over the generations (*P* < 0.05, Mann-Kendall trend test). These genes can be clustered into two categories: the gradually up-regulated and down-regulated genes (Figure 2D). Increasing the number of clusters to 4 revealed the same trend (Supplementary Figure 1), showing the robustness of the clustering analysis. The GO-Slim enrichment analysis showed that the gradually up-regulated genes were mainly enriched in respiration (energy production) and ribosome, while the coenzyme biosynthetic process and drug metabolism process. This result hinted that the translation may be enhanced as the major response to the antibiotics.

### tRNA Up-Regulation During the Subculture Provided Antibiotic Resistance

**FIGURE 3:**
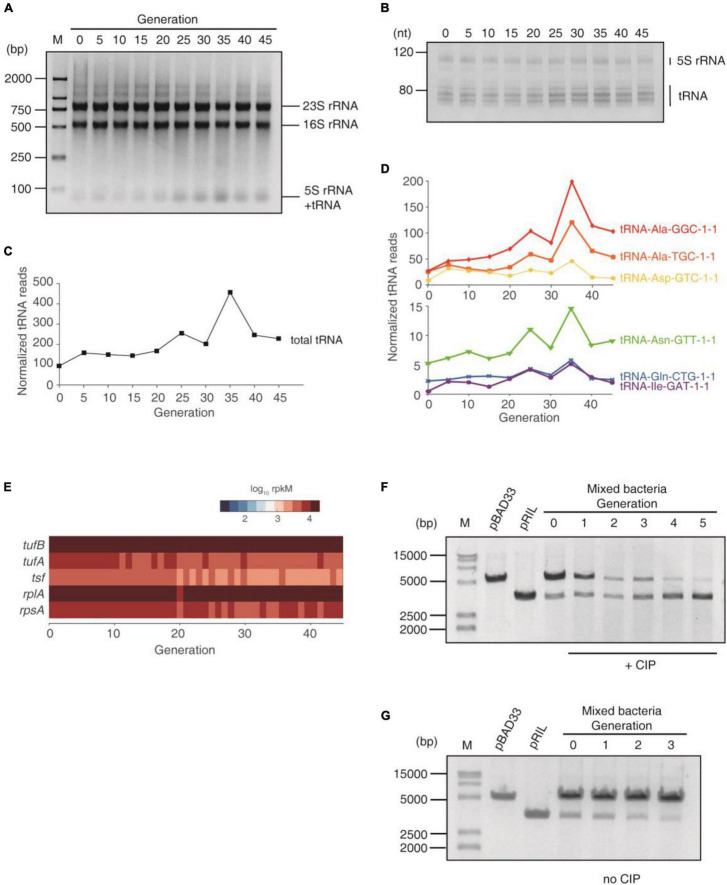
tRNA up-regulation under the CIP stress. **(A)** Agarose gel of total RNA of each generation. Equal amounts of total RNA was loaded. **(B)** Polyacrylamide gel of total RNA of each generation. **(C)** Total tRNA abundance of each generation, quantified using tRNA-seq, normalized using artificially synthesized tRNA spike-in. **(D)** The abundance of 6 most abundant tRNAs during the subculturing, normalized using the spike-in. **(E)** The expression of elongation factors and ribosomal proteins. **(F,G)** The competition assay. pBAD33 and pRIL plasmids were transformed into *E. coli* BW25113, respectively, and mixed to start subculturing. For each generation, the plasmids were extracted from the mixture. **(F)** Subculture with CIP. **(G)** Subculture without CIP.

We loaded equal amounts of total RNA of each generation on gels. On agarose gel, the 23S and 16S rRNA bands were equal in all generations. However, the 5S rRNA + tRNA band gradually increased until the 35th generation (Figure 3A). In polyacrylamide gel, which resolved 5S rRNA and tRNAs, the 5S rRNA were equal in all generations, and the tRNA bands increased (Figure 3B). To investigate the up-regulation of tRNAs, we then performed tRNA-seq using artificially synthesized tRNA as spike-in to quantify the tRNA levels. The tRNA reads were normalized against the spike-in. The result showed that the total tRNA increased during the entire subculture, even at the early stage (Figure 3C), which reproduced the trend on gels. Moreover, most tRNA species were up-regulated at an early stage (Figure 3D), indicating that many tRNAs were affected. In contrast, the translation elongation factors (*tufA/tufB* for EF-Tu and *tsf* for EF-Ts) and the ribosomal proteins (e.g., *rplA* for large ribosomal unit and *rpsA* for small ribosomal unit) were not increased over the entire subculturing, at least on the transcription level (Figure 3E). Together with the constant rRNA content (Figures 3A,B), these results demonstrated that the translational machinery was not up-regulated. Therefore, tRNA up-regulation would effectively maintain the translational elongation and thus help the survival under the ROS induced by CIP at an early stage.

To further examine the significance of tRNA upregulation at early stages of AR, we performed the competition experiment. We transformed the pRIL (which expresses three tRNAs) and pBAD33 (which does not contain any tRNA, serving as a negative control) plasmids into *E. coli* BW25113 strain, respectively. This pair of plasmids were used in previous studies to investigate the effect of tRNA up-regulation (Zhang et al., 2009; Fedyunin et al., 2012; Zhong et al., 2015). We mixed these two types of bacteria together and started subculturing with and without sub-lethal concentration of CIP in the same tube. For each generation (12 h culture), the plasmids of the mixed bacteria were extracted and resolved on agarose gel. With CIP culture, the pRIL plasmid became dominating after 3 generations, and the pBAD33 almost diminished after 5 generations, demonstrating that the excessive tRNA provides growth advantage under antibiotics (Figure 3F). In contrast, without CIP, the pRIL-containing bacteria cannot compete with the pBAD33-containing counterparts, and the pRIL plasmid almost diminished after 3 generations (Figure 3G). This coincides with our previous studies: under the normal conditions, the excessive tRNAs will suppress translational pausing and thus lead to massive protein misfolding and aggregation (Zhang et al., 2009; Fedyunin et al., 2012; Zhong et al., 2015). Under the ROS (generated by CIP), the translation elongation is globally decelerated, and thus the translational pausing is not an issue. Up-regulated tRNAs will maintain the protein production to counteract the oxidative lesion (Zhong et al., 2015). In sum, these results demonstrated that tRNA up-regulation provided survival competence under the antibiotics by maintaining translation activity.

### Genome Recombination Is Enriched Near tRNA Genes

**FIGURE 4:**
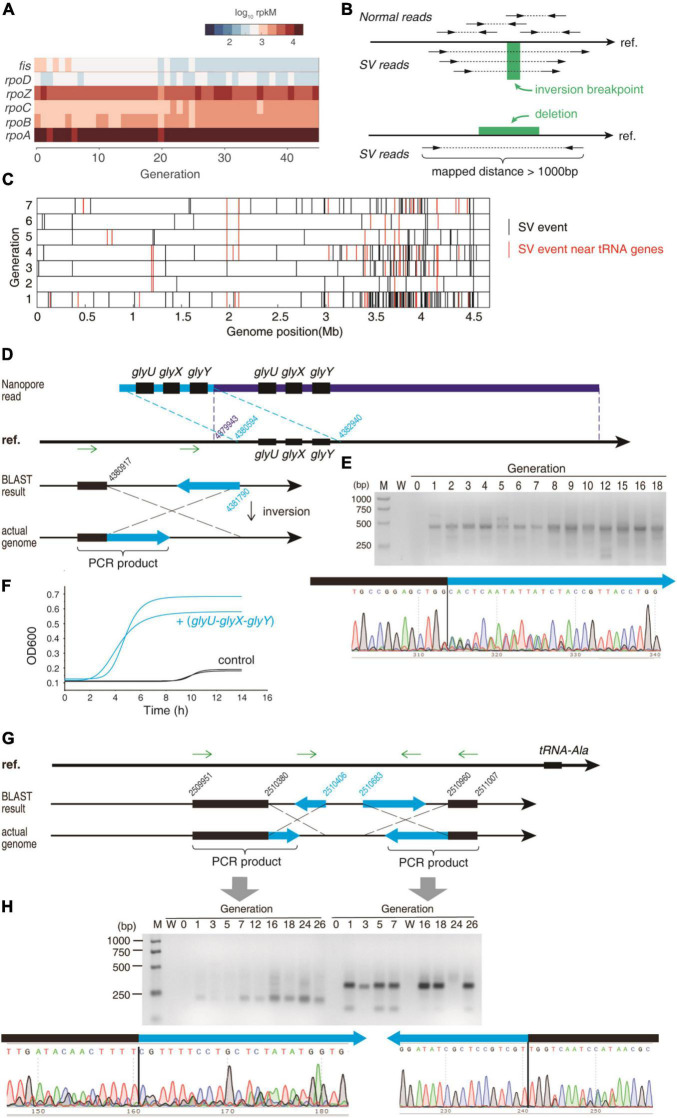
The SV that affects the tRNAs. **(A)** The expression levels of the major transcription factors that are involved in tRNA expression. **(B)** Detecting SV events using paired-end reads. The WGS library was constructed as ∼300 bp insert. After mapped to the reference genome sequence (“ref.”), the two ends of the same read should be mapped in the opposite direction. An inversion breakpoint candidate is identified if the two ends of the same read are mapped in the same direction. Deletion of a fragment is detected if the two ends of the same read are mapped in the opposite direction but far apart. Only the uniquely mapped reads were taken into consideration. **(C)** The SV events were detected using WGS data during the early stage. Red bars represent the SV events near the tRNA genes, and the black bars represent the other events. **(D)** An example of a duplication event and an inversion event near *glyU-glyX-glyY* operon. **(E)** The gel electrophoresis of the PCR product validating the inversion event in **(D)**. The PCR was performed using the two primers that are indicated as green arrows in **(D)**. Lane M = marker; lane W = water control (negative control using water as template). The Sanger sequencing of the PCR product is exhibited for the junction of the inversion. **(F)** Growth curve of the BW25113 expressing the *glyU-glyX-glyY* tandem repeat and the control strain under the CIP stress at 1/2 MIC of the sensitive strain. Growth curves were measured as two independent biological replications. **(G)** Two inversion events near the tRNA-Ala gene. **(H)** The gel electrophoresis of the PCR product validating the inversion events in **(G)**. The PCR was performed using the primers that are indicated as green arrows in **(G)**. Lane M = marker; lane W = water control (negative control using water as template). The Sanger sequencing of the PCR products are exhibited for the junctions of the inversion.

Next, we need to explain the potential reason of tRNA up-regulation upon CIP pressure. We first checked the expression levels of the transcription factors of tRNA genes. However, the transcription factor *fis* for tRNA genes and the other major transcription factors were not increased (Figure 4A). The *E. coli* tRNA genes are transcribed in operons, started from promotors. However, no point mutation was discovered in or near the tRNA genes. Nevertheless, point mutation is not necessary for tRNA up-regulation. If fused into other operons, the tRNA gene will be expressed under the control of that operon, and can be expressed more than 10-fold than the wild-type, which has been observed in the literature (Murakawa and Nierlich, 1987). CIP kills bacteria by causing DNA double-strand breakage via producing ROS and targeting gyrA/gyrB (van der Putten et al., 2019). Such DNA lesion will be repaired primarily by the recombination system, which often leads to genome shuffling and thus leads to rapid phenotypic improvement (Zhang et al., 2002). Therefore, we postulated that genome recombination may have happened near tRNA genes to interfere with tRNA expression.

To test this hypothesis, we analyzed the structural variation (SV) of the bacterial genome from the WGS paired-end sequencing data (Figure 4B). From the first generation after applying CIP, many SVs were detected, including deletions and inversions (Figure 4C). In the first 7 generations, 341 SVs were detected, and 63 SVs were detected near the tRNA genes (defined as ±3,000 nt near tRNA genes). The actual probability that an SV occurs near tRNA genes was significantly higher than the random distribution (*P* = 4.74 × 10^–35^, Fisher Exact Test), suggesting that the tRNA genes and their flanking sequences are more susceptible to the double-strand break induced by antibiotics.

Next, we validated some SV events near the tRNA genes in the 4th generation. We subjected the high-quality genomic DNA (OD260/280 = 1.8, 36.5 ng/μl, 1 μg used) to Nanopore sequencing to obtain long reads. We found 1408 SV events in the nanopore sequencing dataset, and 57 among them were located near the tRNA genes (*P* = 8.97 × 10^–6^, Fisher exact test), suggesting that the SVs occur significantly more often near tRNA genes. This validated the MGISEQ-2000 results. We found a long read that contained two sets of glycine tRNAs (*glyU-glyX-glyY*) (Figure 4D). This 23,394 nt read was aligned to reference genome as two segments, demonstrating a duplication that multiplicated tRNA copy number.

An inversion event was also found in the upstream of the *glyU-glyX-glyY* operon at 4,380,917 supported by 41 paired-end reads (Supplementary Figure 2A). Using two primers in the same direction (designed according to the reference genome), no PCR result could be yielded unless an inversion occurs (Figure 4D). Indeed, a band of 415 bp PCR product was found (Figure 4E). After sanger sequencing, the fragment can be aligned to the reference genome in two segments in opposite direction, demonstrating an inversion from 4380,917 to 4381,790 (Figure 4D). Coincidently, the tRNA-Gly-GCC, which is transcribed from the *glyU-glyX-glyY* operon, was found increased in the ciprofloxacin stress (Supplementary Figure 3). Two inversions were detected in the upstream sequence of tRNA-Ala: 2,510,380–2,510,406 and 2,510,683–2,510,960 (Figure 4G). These two events were also validated using the abovementioned PCR method (Figure 4H). Coincidently, the alanine tRNA was upregulated in the ciprofloxacin stress (Figure 3D). A similar inversion event near the tRNA-Sec was also validated by PCR and Sanger sequencing. Coincidently, the tRNA-Sec is increased in the 4th generation. Notably, all these PCR evidence showed that these SV events were absent in the original sensitive strain (generation 0), while constantly appear since the first generation under ciprofloxacin stress, indicating that these SV events were induced by antibiotics (Figures 4E,H).

To investigate whether the duplication of the 3-gly-tRNA tandem causes AR at early stage, we synthesized this tandem and replaced the original tRNA genes in the pRIL plasmid. This led to constitutive expression of the 3-gly-tRNA tandem. The pRIL plasmid without tRNA genes were used as negative control (Supplementary Figure 4). Vectors were transformed into wild-type *E. coli* BW25113. The 3-gly-tRNA plasmid created 27 × higher gly-tRNA expression than the control. The MIC of the 3-gly-tRNA tandem overexpressed bacteria is 1.6 × higher than the wild-type sensitive strain. Under the CIP pressure at 1/2 MIC, the 3-gly-tRNA tandem overexpressed bacteria showed approximately 1/5 of the lag phase and raised to OD_600_ = 0.6∼0.7 when it reached the stationary phase (Figure 4F). In contrast, the control strain experienced 9 h lag phase, and eventually reached OD_600_ < 0.2 at the stationary phase. This demonstrated that the 3-gly-tRNA tandem endows remarkable adaptation against antibiotics. This result validated the function of the SV-induced duplicated gly-tRNAs in the AR.

The tRNA-processing enzymes may also be affected by the SV events. For example, the deletion happened between 3,916,925 and 3,917,502, which was detected from the 21st generation, was confirmed by Sanger sequencing (Supplementary Figure 5A) and PCR (Supplementary Figure 5B). Accordingly, the expression of *mnmG* gene was remarkably elevated since the 21st generation (Supplementary Figure 5C). This deletion causes a truncation of the *mnmG* gene. However, it seems that this truncation does not affect the catalytic domain of MnmG enzyme. The nucleotide binding sites of MnmG are all before 370 aa, which is in its main domain, according to the UniProtKB (Supplementary Figure 5D, blue domain). However, the truncation cuts the fragment 497–629 aa, which is in a flexibly linked independent domain 479–629 aa (Supplementary Figure 5D, orange domain). Therefore, the truncation remained an intact main domain, which should maintain its catalytic function. The MnmG is a 5-carboxymethylaminomethyluridine-tRNA synthase. It is responsible for tRNA modification that reduces frameshift errors in translation, according to the EcoCyc database. Therefore, the up-regulation of MnmG may consolidate the protein synthesis quality, which facilitates the stress response.

### Suppressing Recombination Repair System Decreases the Evolution of Intrinsic Antibiotic Resistance

**FIGURE 5:**
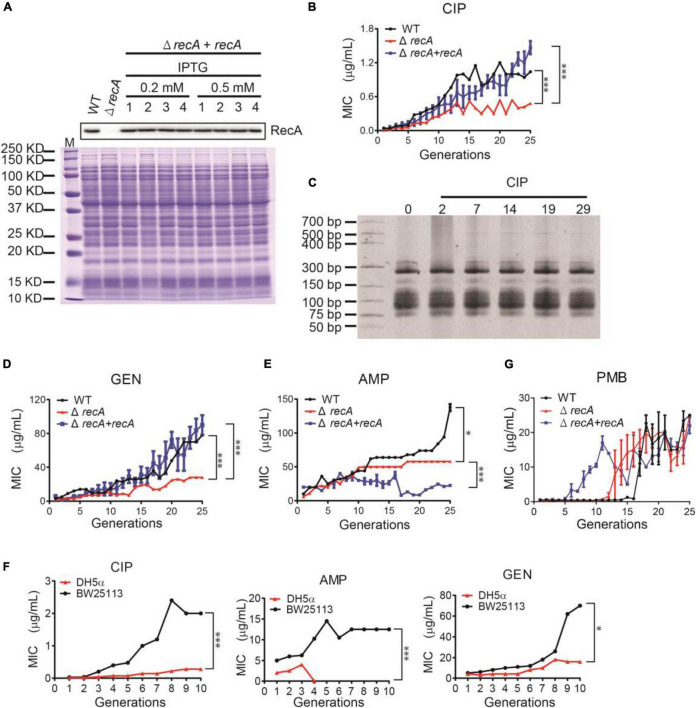
Repressing the recombination abolish the AR evolution. **(A)** The deletion of the *recA* gene (*ΔrecA* strain) and the RecA-restored strain (Δ*recA* + *recA*). Western blot confirmed the RecA expression levels. The total protein gel stained with Coomassie blue was used as loading control for the same loading. 1∼4 represent the 4 independent biological replicates. **(B)** The MIC of the WT (black line), Δ*recA* (red line), and RecA-restored (blue line) strains, respectively, during the subculturing with CIP. **(C)** The tRNA content during the subculturing with CIP, resolved in a PAGE. **(D,E)** The MIC of the three strains during the subculturing with gentamycin (GEN, **D**) and ampicillin (AMP, **E**), respectively. All MICs were illustrated as mean ± SD of three biological replicates. **(F)** The MIC of the WT (black line) and the DH5α strain (red line) during the subculturing with CIP, AMP, and GEN, respectively. *P < 0.05. ***P < 0.0001. **(G)** Similar to **(B,D,E)**, the subculturing of the *E. coli* strains in polymyxin B.

To validate the hypothesis that the recombinant repair causes the tRNA upregulation and thus leads to AR, we suppressed the recombination enzymes by gene knockout. The *E. coli* BW26355 is the Δ*recA* strain (*recA* gene was knocked out from the *E. coli* BW25113 strain). We also cloned the *recA* gene into pET-28b plasmid and transferred to the BW26355 strain to restore RecA expression to the wild-type level (Figure 5A). We subjected these three bacteria to subculture under sub-lethal levels of CIP. As expected, the Δ*rec*A strain evolved AR much slower than the wild-type and the *recA* restored strain. The final MIC that the Δ*recA* strain could reach is also much lower than the other two strains with RecA (Figure 5B). The tRNA content of the Δ*recA* strain did not show a visible increase (Figure 5C). Similar trend also applied to other kinds of antibiotics, such as gentamycin (Figure 5D) and ampicillin (Figure 5E), under which stress Δ*recA* strain evolved AR slower, and showed the maximum MIC lower than the wild-type.

The *E. coli* SOS repair system include four components, namely RecA, LexA, RelA, and EndA. RecA is the most important one, but the other three are not negligible. The *E. coli* DH5α strain lacks all these four recombinases. Therefore, the DH5α strain has minimum recombination ability, much lower than the Δ*recA* strain. Indeed, when subcultured, DH5α can hardly evolve AR (Figure 5F). In the ampicillin-containing media, the DH5α die out after 3 generations. These results showed that the recombination ability is positively correlated to the AR evolution, and this applies to multiple antibiotics.

Interestingly, this mechanism is not (fully) applicable in all types of antibiotics. When subculturing in ampicillin, the *RecA* deletion strain showed significantly lower MIC compared to the wild-type, which matches our theory. However, the *RecA* restored strain could not resume the AR, probably due to other reasons, for example, the insufficient expression in the presence of beta-lactams. When subculturing in polymyxin B, the *RecA* deletion and restored strains showed similar AR like the wild-type after 20∼25 generations (Figure 5G).

## Discussion

**FIGURE 6:**
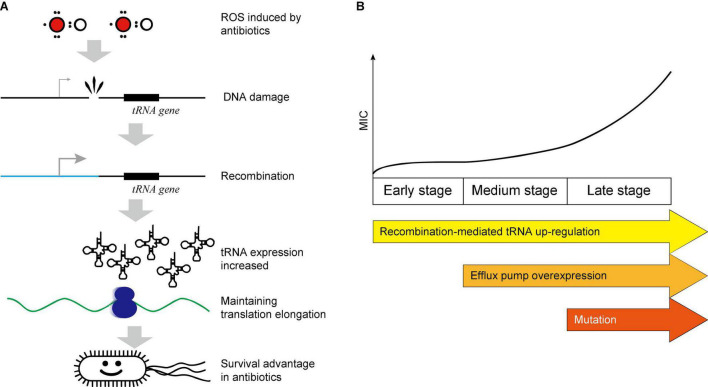
Scheme of the recombination-mediated tRNA up-regulation. **(A)** Pathway illustration of the recombination-mediated tRNA up-regulation. **(B)** Multiple AR mechanisms coordinate in a stepwise manner.

Previous studies on intrinsic AR mainly focused on the mutations, membrane permeability, and the efflux pump up-regulation. However, these three mechanisms need time to accumulate and may need days to evolve (Gullberg et al., 2011; Sass et al., 2011; Toprak et al., 2012). The early stage of the AR evolution, especially the general resistance mechanism against almost all kinds of antibiotics, has been overlooked. Such mechanisms are essential for bacteria to promptly combat the initial attack of antibiotics and thus make time for more specific and efficient resistance to evolve. Here, we provided evidence that the translational response can serve as a general mechanism for AR at early stage. Since most antibiotics induce ROS in bacteria (Kohanski et al., 2007), which induces DNA damage, bacteria start the recombinant repair to response ROS stress which may lead to massive recombination in genomes. The recombination events are enriched near the tRNA genes in bacteria under the constant CIP pressure, probably due to the highly homologous sequences (Williams, 2002; Chan and Lowe, 2016). This will, by chance, elevate the tRNA expression, and thus maintain the translational efficiency to synthesize enough proteins to maintain physiological processes, fix the impaired components, and counteract the stress (Zhong et al., 2015), which is essential for the survival (Zhu and Dai, 2019), especially at the early stage, and thus protect the cell from the destruction (Figure 6A).

As a general mechanism, the recombination and tRNA-mediated general AR at an early stage do not target specific antibiotics, but rather apply to the antibiotics which induce ROS. It can be explained that the ROS is a major cause of DNA damage and thus induces the recombination. The antibiotics CIP, GEN, and AMP induce ROS to different extents, and thus can be counteracted by tRNA up-regulation. The antibiotics used in Hoeksema et al. (2018) (AMX, ENRO, KAN, TET) belong also to the same category, and they observed genome rearrangements in the *de novo* acquisition of AR. However, their study did not reveal the mechanism how the rearrangements could lead to AR. Since most antibiotics induce ROS, this mechanism is sufficient for the survival in most cases. Indeed, we also showed that the polymyxin B, which does not generate ROS at sub-MIC concentration (Lima et al., 2019), cannot be resisted by this mechanism (Figure 5G). This again suggested that the tRNA-mediated AR applies only on the ROS-generating antibiotics.

To be noted, multiple AR mechanisms are coordinated and are involved sequentially in a stepwise manner. At the early stage, the tRNA up-regulation provided a prompt protection at an early stage. From the 8th generation, the efflux pump expression was increased. From the 22nd generation, the genomic mutation provided effective resistance at the late stage (Figure 6B). This coincides with the timescale of the regulation happens at different levels: the translation regulates most quickly, the transcription reacts slower, and the genomic mutations usually need prolonged time to accumulate. However, the effect size is *vice versa*. We previously reported that the tRNA up-regulation efficiently counteracts the ROS generated by hydrogen peroxide within 90 min by maintaining translation processes (Zhong et al., 2015), demonstrating that the translational response can provide the initial protection against antibiotics. After the full adaptation and evolution of high AR by mutations, the tRNA overexpression is not necessary. Therefore, the tRNA reaches maximum at ∼35 generations and then decreases (Figures 3B–D).

Although the excessive tRNA provides advantages in the presence of antibiotics, they must be tightly controlled in the absence of antibiotics. Under normal conditions, excessive tRNA reduces the translational pausing and leads to massive misfolding, creating additional stress (Zhang et al., 2009; Fedyunin et al., 2012) and thus reduces the fitness (Zhong et al., 2015). In our competition experiment, the tRNA elevated strain showed disadvantage under normal conditions. However, in the presence of antibiotics, the ROS slows down the translation in general. Therefore, elevating tRNA helps to maintain translational elongation rate is beneficial for the survival (Zhong et al., 2015; Zhu and Dai, 2019).

Interestingly, the tRNA genes and their flanking sequences seems to be sensitive to the DNA damage and recombination. It has been shown that the tRNA genes serve as high frequency integration sites for genetic elements in prokaryote genomes (Williams, 2002). The highly similar and conserved sequences and small size favors the recognition of integration sites. Also, these regions are also favorable to horizontal gene transfer (Oliveira et al., 2017). However, these previous studies did not investigate the recombination around the tRNA genes and the influence of tRNA expression. Maintaining the translational elongation rate does not require overexpression of all tRNAs. This largely increases the probability of successful “resistance recombination.” Random elevation of several tRNAs is enough to provide survival advantage in the presence of antibiotics, which has been validated using the pRIL plasmid (Figures 3F,G, 4D–F). Moreover, it has been reported that the bacterial tRNA genes are neither evenly nor randomly distributed in genomes (Fan et al., 2007; de Bruijn et al., 2012). This distribution pattern might be also linked to the high frequency of recombination near tRNA genes.

Finally, our study provided a hint to suppress the evolution of intrinsic AR: suppressing the bacterial recombination systems while applying antibiotics. This can effectively delay or even abolish the emergence of resistance against most antibiotics. Since RecA is one of the most important recombinases (Galletto and Kowalczykowski, 2007), and it is highly conserved in almost all eubacteria, both in sequence and in structure (Miller and Kokjohn, 1990), a small molecule which binds and blocks RecA of many bacterial species might be feasible. Although the risk of acquired AR by horizontal transfer or resistance genes still exists, this reduces the risk of intrinsic resistance, especially when using a newly artificial antibiotic, for which the resistance gene does not yet exist.

## Data Availability Statement

The datasets presented in this study can be found in online repositories. The names of the repository/repositories and accession number(s) can be found in the article/Supplementary Material.

## Author Contributions

GoZ contributed to conception. GoZ and XS designed the study. HF, GuZ, WG, YW, JZ, TZ, LX, YL, and JNZ performed the experiments. HF, WG, YW, JZ, and TZ analyzed the data. HF, WG, YW, XS, and GoZ wrote the manuscript. All authors contributed to manuscript revision, read, and approved the submitted version.

## Conflict of Interest

The authors declare that the research was conducted in the absence of any commercial or financial relationships that could be construed as a potential conflict of interest.

## Publisher’s Note

All claims expressed in this article are solely those of the authors and do not necessarily represent those of their affiliated organizations, or those of the publisher, the editors and the reviewers. Any product that may be evaluated in this article, or claim that may be made by its manufacturer, is not guaranteed or endorsed by the publisher.

## Footnotes

1
http://www.chi-biotech.com/technology.html?ty=ypt

2
http://www.pantherdb.org/

3
http://gtrnadb.ucsc.edu/
