# Supplementary material for: Genome Recombination-Mediated tRNA Up-Regulation Conducts General Antibiotic Resistance of Bacteria at Early Stage
Source: Front Microbiol. 2022 Jan 20;12:793923. doi: 10.3389/fmicb.2021.793923 (PMC8811037; doi:10.3389/fmicb.2021.793923)

SUPPLEMENTARY MATERIALS

Table S1: Summary of the WGS of each generation.

Generation	Read count	Total nucleotides (Gb)	Mapping%	Average depth (x)	Percentage of >=10x regions
0	8,810,400	881.04	97.70%	187.46	99.05%
1	13,764,011	1376.40	98.10%	292.85	99.05%
2	9,017,907	901.79	94.50%	191.87	99.04%
3	9,212,062	921.21	98.00%	196.00	99.04%
4	8,613,258	861.33	97.90%	183.26	99.05%
5	8,073,276	807.33	97.80%	171.77	99.04%
6	18,875,781	1887.58	98.10%	401.61	99.05%
7	20,196,632	2019.66	86.70%	429.72	99.05%
8	4,503,135	450.31	98.10%	95.81	99.03%
9	5,376,502	537.65	98.10%	114.39	99.03%
10	9,297,243	929.72	98.10%	197.81	99.05%
11	20,667,315	2066.73	98.10%	439.73	99.05%
12	16,140,953	1614.10	98.10%	343.42	99.05%
13	9,315,677	931.57	83.20%	198.21	99.04%
14	10,178,850	1017.89	87.90%	216.57	99.04%
15	11,536,580	1153.66	97.80%	245.46	99.05%
16	14,386,801	1438.68	97.60%	306.10	99.05%
17	12,381,226	1238.12	96.10%	263.43	99.05%
18	32,611,631	3261.16	98.30%	693.86	99.05%
19	10,659,337	1065.93	98.00%	226.79	99.05%
20	9,627,783	962.78	96.70%	204.85	99.05%
21	14,283,937	1428.39	98.00%	303.91	99.05%
22	11,183,570	1118.36	92.00%	237.95	99.05%
23	12,073,814	1207.38	98.00%	256.89	99.05%
24	12,050,429	1205.04	97.90%	256.39	99.05%
25	24,878,208	2487.82	98.10%	529.32	99.05%
26	18,767,112	1876.71	98.00%	399.30	99.05%
27	16,503,457	1650.35	97.90%	351.14	99.05%
28	22,084,659	2208.47	98.00%	469.89	99.05%
29	18,396,227	1839.62	98.10%	391.41	99.05%
30	20,220,073	2022.01	96.40%	430.21	99.05%
31	22,557,079	2255.71	89.70%	479.94	99.05%
32	16,819,259	1681.93	98.00%	357.86	99.05%
33	25,595,880	2559.59	98.10%	544.59	99.05%
34	23,207,842	2320.78	98.00%	493.78	99.07%
35	17,124,280	1712.43	98.40%	364.35	99.05%
36	3,710,807	371.08	96.40%	78.95	99.01%
37	14,956,087	1495.61	98.30%	318.21	99.05%
38	18,088,530	1808.85	97.60%	384.86	99.05%
39	20,478,372	2047.84	97.30%	435.71	99.06%
40	3,132,407	313.24	93.40%	66.65	98.98%

41	8,625,582	862.56	95.70%	183.52	99.04%
42	10,342,404	1034.24	96.70%	220.05	99.03%
43	11,303,880	1130.39	96.10%	240.51	99.03%
44	11,408,086	1140.81	95.90%	242.73	99.03%
45	11,757,863	1175.79	96.20%	250.17	99.04%

Table S2: SNV list (see Fig. 1C)

Type	Position	Mutation	CDS ID	WP ID	Gene	Product
**CDS**	481432	A>C	cds486	WP_000101737.1	*acrR*	transcriptional regulator
	3871241	G>A	cds3922	WP_000072067.1	*gyrB*	DNA gyrase subumit B
	3916926	T>G	cds3965	WP_000932839.1	*rsmG*	ribosomal RNA small subumit methyitransferase G
	3916927	G>A				
	3917493	C>A	cds3966	WP_000499788.1	*mnmG*	tRNA uridine(34) 5-carboxymethyl-aminomethyl synthesis enzyme
	4174942	A>T	cds4194	WP_000263098.1	*rpoB*	DNA-directed RNA polymerase subunit beta
**non-coding**	1972746	C>T				

Figure S1: The K-means clustering with 4 clusters in the trend analysis of the transcriptome over the time (see Fig. 2D). Basically, the cluster 2+4 here correspond to the Cluster 1 in Fig. 2D, and the cluster 1+3 correspond to the Cluster 2 in Fig. 2D.

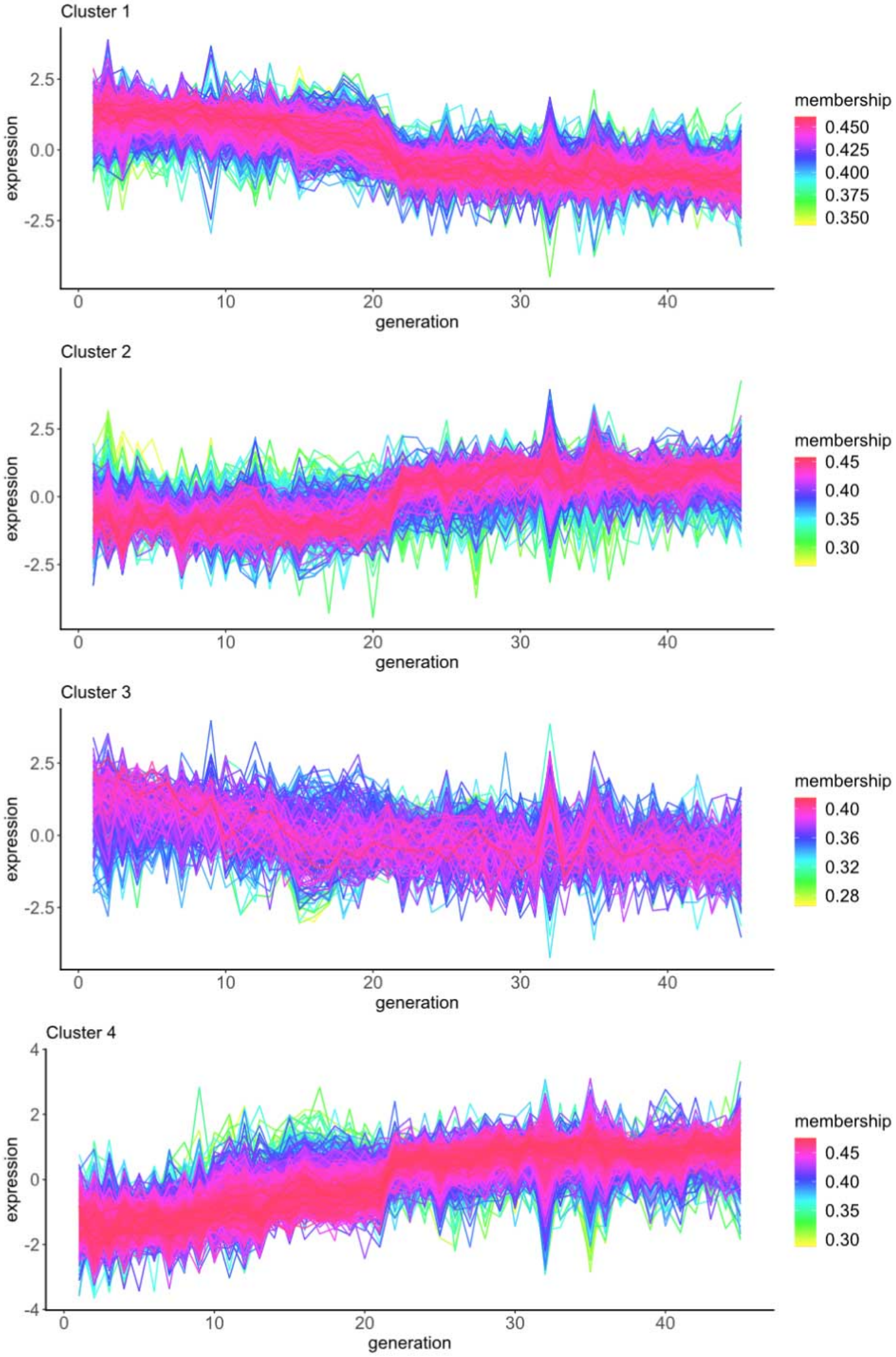

Figure S2: MGISEQ-2000 reads that supports inversion events near tRNA genes illustrated in Fig. 4D~G. Red dashed line denotes the inversion junction point. Genome positions represent the *E. coli* BW25113 reference genome coordinate. Each horizontal line denotes one paired-end read. Arrows illustrates the mapped position of both ends and the mapped direction (right = mapped to forward strand; left = mapped to reverse strand). (A) The inversion junction in Fig. 4D~E. (B) The inversion junction in Fig. 4F~G.

A

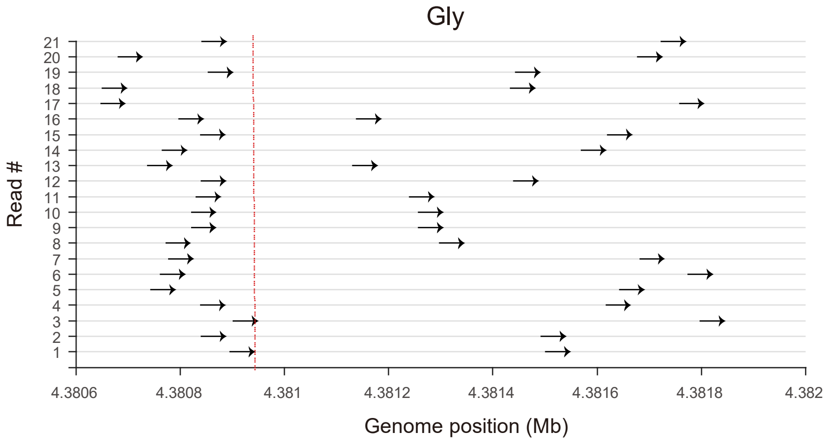

B

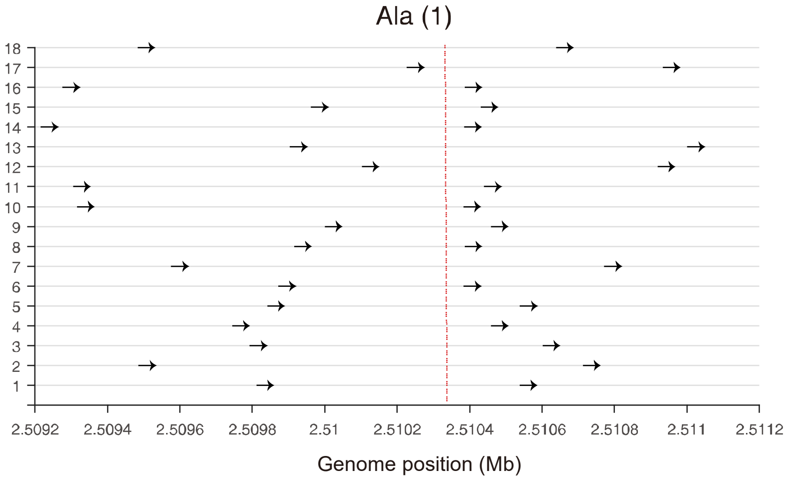

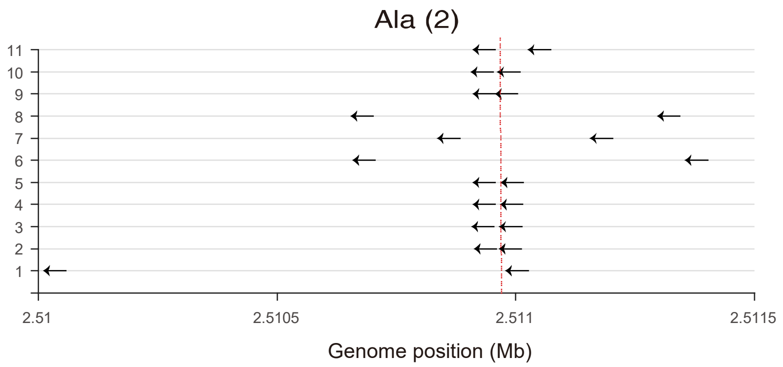

Figure S3: Up-regulation of tRNA-Gly-GCC-1-1 during subculturing.

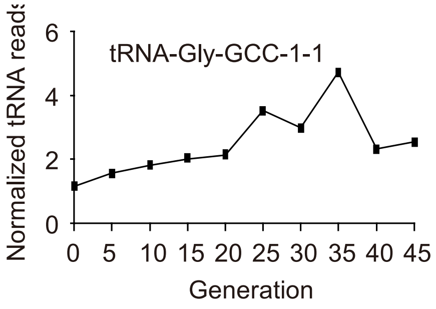

Figure S4: Vectors costruction. Synthesized 3-gly-tRNA tandem was constructed into the red segment of pRIL plasmid, replaced the origin tRNA genes in the pRIL. The pRIL plasmid without tRNA genes were used as negative control.

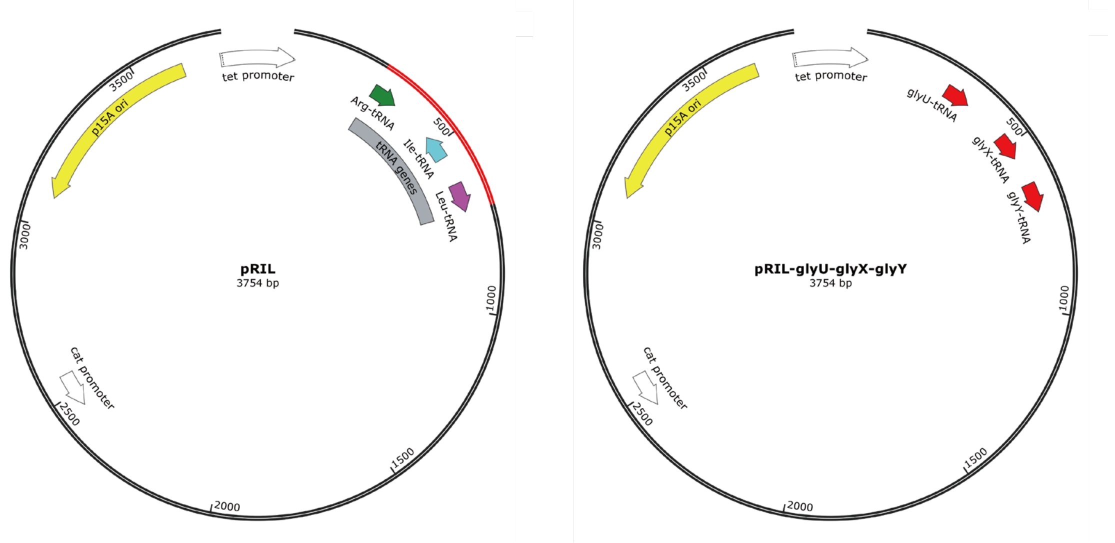

Figure S5: An example of a deletion which started at the 21st generation. This deletion affected *mnmG* gene encoding tRNA-processing enzyme. (A) Position of the deletion and the Sanger sequencing validation. (B) PCR validation using primers in the flanking sequences. The "original" mark denotes the expected band if there's no deletion, and the "deleted" mark denotes the expected size of the product with deletion. (C) The expression level of the *mnmG* gene in the RNA-seq data. (D) The structure of MnmG protein (PDB: 3CP2). Blue and green domains are two domains according to the CATH Protein Structure Classification Database v4.3 (https://www.cathdb.info/). Blue domain is the catalytic domain which binds nucleic acids. The truncated fragment caused by the deletion is marked as light orange color.

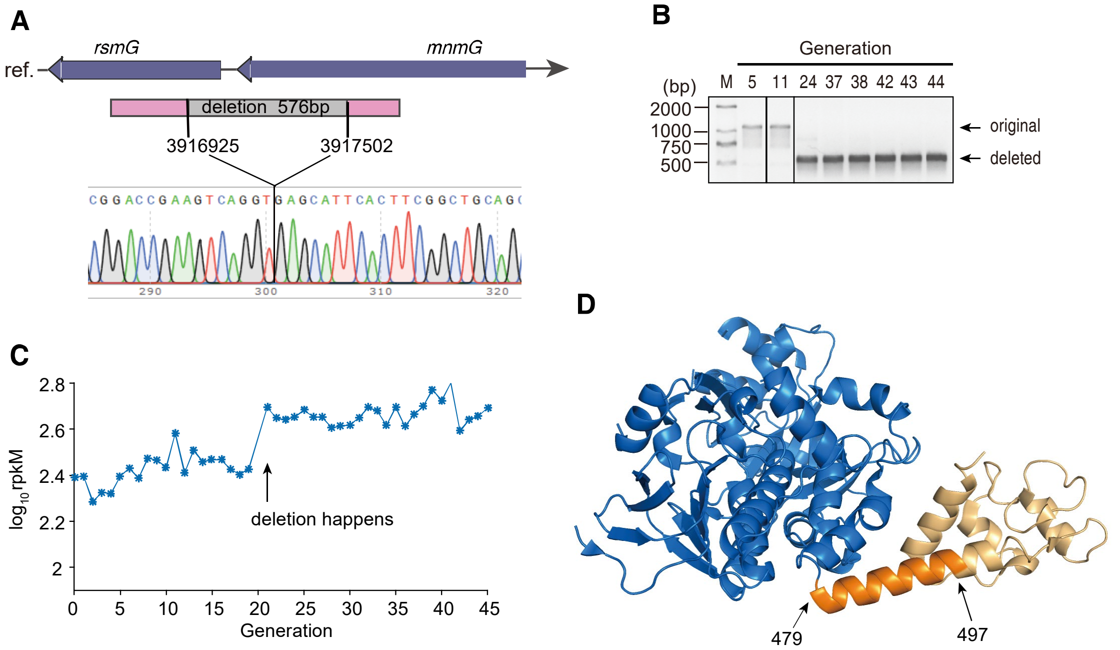

Supplement: Supplementary file 1 [file Data_Sheet_1.PDF]
